# Upgrading syngas fermentation effluent using *Clostridium kluyveri* in a continuous fermentation

**DOI:** 10.1186/s13068-017-0764-6

**Published:** 2017-03-29

**Authors:** Sylvia Gildemyn, Bastian Molitor, Joseph G. Usack, Mytien Nguyen, Korneel Rabaey, Largus T. Angenent

**Affiliations:** 1 Cornell University, Biological and Environmental Engineering, Riley-Robb Hall, Ithaca, NY 14853 USA; 2 Ghent University, Center for Microbial Ecology and Technology (CMET), Coupure Links 653, 9000 Ghent, Belgium; 3 University of Tübingen, Center for Applied GeoSciences, Hölderlinstr. 12, 72074 Tübingen, Germany; 4 Organic Waste SystemsDok Noord 5, 9000 Ghent, Belgium

**Keywords:** Chain elongation, Carboxylate platform, Syngas fermentation, Carboxylic acids, *n*-Caproic acid, *n*-Caprylic acid

## Abstract

**Background:**

The product of current syngas fermentation systems is an ethanol/acetic acid mixture and the goal is to maximize ethanol recovery. However, ethanol currently has a relatively low market value and its separation from the fermentation broth is energy intensive. We can circumvent these disadvantages of ethanol production by converting the dilute ethanol/acetic acid mixture into products with longer carbon backbones, which are of higher value and are more easily extracted than ethanol. Chain elongation, which is the bioprocess in which ethanol is used to elongate short-chain carboxylic acids to medium-chain carboxylic acids (MCCAs), has been studied with pure cultures and open cultures of microbial consortia (microbiomes) with several different substrates. While upgrading syngas fermentation effluent has been studied with open cultures, to our knowledge, no study exists that has performed this with pure cultures.

**Results:**

Here, pure cultures of *Clostridium kluyveri* were used in continuous bioreactors to convert ethanol/acetic acid mixtures into MCCAs. Besides changing the operating conditions in regards to substrate loading rates and composition, the effect of in-line product extraction, pH, and the use of real syngas fermentation effluent on production rates were tested. Increasing the organic loading rates resulted in proportionally higher production rates of *n*-caproic acid, which were up to 40 mM day^−1^ (4.64 g L^−1^ day^−1^) at carbon conversion efficiencies of 90% or higher. The production rates were similar for bioreactors with and without in-line product extraction. Furthermore, a lower ethanol/acetic acid ratio (3:1 instead of 10:1) enabled faster and more efficient *n*-caproic acid production. In addition, *n*-caprylic acid production was observed for the first time with *C. kluyveri* (up to 2.19 ± 0.34 mM in batch). Finally, the use of real effluent from syngas fermentation, without added yeast extract, but with added defined growth factors, did maintain similar production rates. Throughout the operating period, we observed that the metabolism of *C. kluyveri* was inhibited at a mildly acidic pH value of 5.5 compared to a pH value of 7.0, while reactor microbiomes perform successfully at mildly acidic conditions.

**Conclusions:**

*Clostridium kluyveri* can be used as a biocatalyst to upgrade syngas fermentation effluent into MCCAs at pH values above 5.5.

**Electronic supplementary material:**

The online version of this article (doi:10.1186/s13068-017-0764-6) contains supplementary material, which is available to authorized users.

## Background

The production of biochemicals as alternatives to fossil fuel-based chemicals is gaining momentum. Increasing awareness that the use of fossil fuels leads to climate change and air pollution has resulted in the development of several bioproduction routes [1]. First, with the sugar platform, the use of starch and sugar crops to produce bioethanol currently results in production rates exceeding 60 g L^−1^ day^−1^ with commercial bioproduction strains [2]. More than 100 million tons of bioethanol are produced worldwide every year [3]. This platform is controversial because crop cultivation requires a high input of land, water, and nutrients [4]. Cultivation of these crops for biofuel production may also compete with the production of food. Lignocellulosic biomass as feedstock circumvents these issues but requires addition of chemicals and process energy to pretreat the biomass to make the sugars available for fermentation, while a large percentage of the biomass remains unused [1].

Second, with the syngas platform, catalytic conversion of lignocellulosic biomass to syngas (H_2_/CO/CO_2_ gas mixtures), and subsequent autotrophic fermentation of these gases to liquid products, could enable high carbon efficiency while still avoiding crop-related concerns [5]. The syngas platform has potential because, in addition to lignocellulosic biomass and other organic waste streams, point sources of syngas are widely available such as the off-gases from steel manufacturing [6–8]. The use of industrial gaseous off streams can lead to decreased greenhouse gas emissions coupled to the production of multicarbon products via fermentation, with ethanol currently being the dominant product. Anaerobic autotrophic bacteria, such as *C. ljungdahlii* and *C. autoethanogenum*, carry out this bioconversion. These bacteria utilize the Wood–Ljungdahl pathway for carbon fixation [9, 10]. The usual product of the autotrophic metabolism is acetic acid. When the intracellular, undissociated acetic acid concentration reaches a threshold, the metabolism is redirected towards ethanol production to decrease toxicity and increase the number of reducing equivalents per mole of product formed [11, 12]. Ethanol production rates exceeding 200 g L^−1^ day^−1^ are now obtainable with commercial strains [6].

Third, with the carboxylate platform, treatment of organic wastes is coupled to bioproduction via anaerobic fermentation processes. The carboxylate platform primarily involves the production of short-chain carboxylic acids (SCCAs) and gases, such as H_2_ and CO_2_, by utilizing open cultures of microbial consortia (microbiomes) [13, 14]. Inhibition of methanogenesis, which is the last step in the anaerobic foodweb for biogas production, results in the buildup of SCCAs in the fermentation broth. Chain elongation via the reverse β-oxidation pathway then leads to the production of medium-chain carboxylic acids (MCCAs) within the microbiome [15, 16]. Among the end products, *n*-caproic acid and *n*-caprylic acid are two desirable MCCA bioproducts, but they are characterized by high product toxicities at mildly acidic pH values (pKa ~4.8) [17]. Circumvention of product toxicity and high-rate fermentation can be achieved by the following: (i) continuous removal of the produced carboxylic acids via in-line extraction; (ii) applying high dilution rates; or (iii) maintaining a favorable pH for acid dissociation [18, 19]. At high dilution rates and neutral pH, the maximum *n*-caproic acid production rate of 55 g L^−1^ day^−1^ has been achieved [20].

**Fig. 1:**
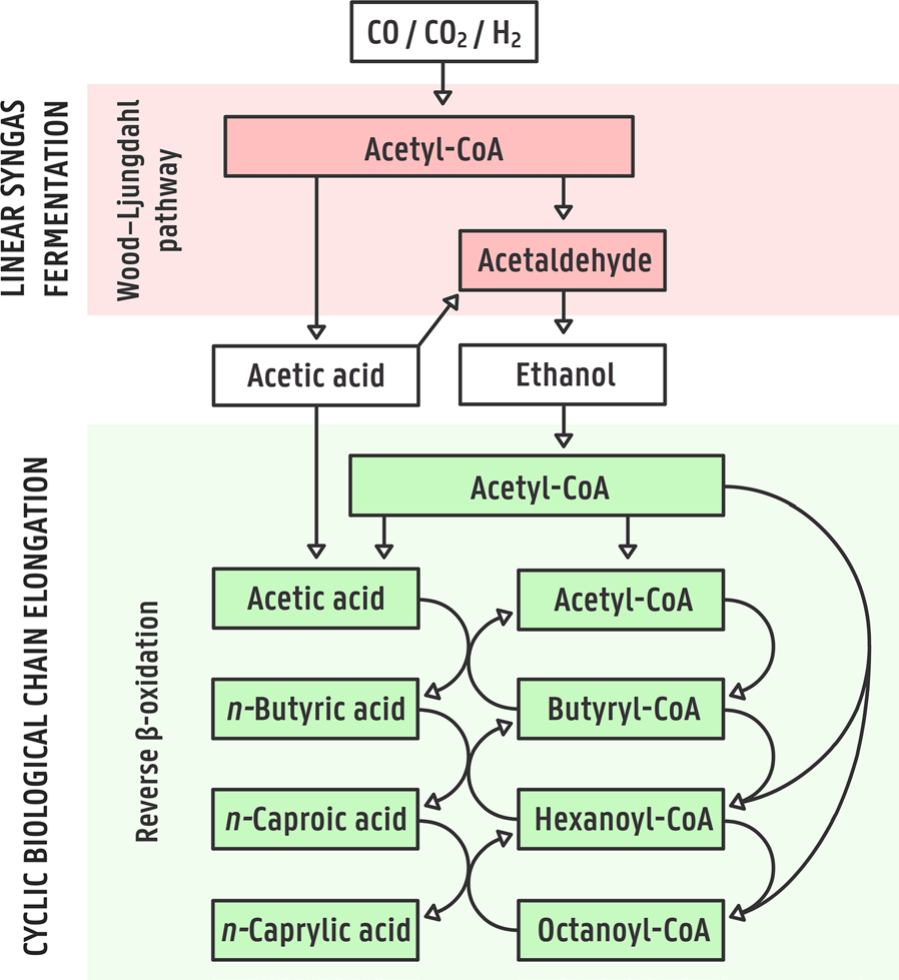
Simplified pathways showing the coupling of syngas fermentation, relying on the Wood–Ljungdahl pathway, and biological chain elongation, relying on reverse β-oxidation. The* red *and* green box* represents the boundary of the microorganism performing the pathway. More details on both pathways can be found in References [8] and [42]

Fourth, coupling different production platforms to upgrade side streams or produce higher value chemicals could create additional value. For example, syngas fermentation only leads to a marketable product when the fully miscible ethanol is extracted and separated from the fermentation broth. Distillation of dilute ethanol (6% w/v in syngas fermentation effluent) is an energy-intensive process that results in a currently low-value product. Mixtures of ethanol and acetic acid in syngas fermentation effluent, in particular those with high ethanol/acetic acid ratios, could also be further biologically converted to MCCAs via chain elongation (Fig. 1) [21–23]. One of the main questions is whether growth factors, such as trace elements or yeast extract, would have to be added to the syngas fermentation effluent to enable the chain elongation process, or if those already present in the effluent would suffice to support growth. Addition of growth factors would greatly increase the production cost for MCCAs. In two proof-of-concept studies that coupled chain elongation to syngas fermentation, several growth factors, such as yeast extract, were added along with the substrate. The necessity of these additions was, however, not studied [21, 22].

The chain elongation process and its underlying thermodynamics are now well understood [23–25]. Several microorganisms are known to convert ethanol/acetic acid mixtures via the reverse β-oxidation pathway, with *C. kluyveri* being the most frequently studied microbe. *C. kluyveri* has the ability to produce *n*-butyric and *n*-caproic acid from ethanol and acetic acid [26], and has regularly been found in microbiomes that converted either synthetic or real organic waste streams into MCCAs [18, 27]. Except for one study that determined substrate utilization, pure-culture continuous bioreactor studies with *C. kluyveri* are lacking [28]. Pure-culture fermentation would allow more control of the chain elongation process. Furthermore, this would enable the investigation of different process parameters and their influence on the productivity of the culture. From studies of thermodynamic models and pure and open cultures it is known: (i) a higher ethanol/acetic acid ratio drives the chain elongation reaction towards longer carbon chain carboxylic acids, including *n*-caprylic acid [22, 23, 29]; and (ii) selective in-line extraction of longer chain products results in higher product specificities [23, 30]. Currently, microbiome studies are achieving high production rates of *n*-caprylic acid [22], but this product has not yet been detected in pure *C. kluyveri* studies.

To address the aforementioned knowledge gaps, we have conducted experiments using a pure culture of *C. kluyveri* in bioreactors to investigate whether syngas fermentation effluent can be directly used as a growth medium for *C. kluyveri*. We report for the first time *n*-caprylic acid production by a pure culture of *C. kluyveri*. In addition, we elaborate on key challenges to obtain high-rate MCCA production from syngas fermentation effluent in bioreactors with and without in-line product extraction. In the context of this article “carboxylic acids” is used as the general term for the products obtained. Depending on the pH of the processes described, these carboxylic acids are fully or partially present in their dissociated forms.

## Methods

### Culture and media

A *Clostridium kluyveri* ATCC 8527 (DSM555) culture was obtained from ATCC (Manassas, VA, USA) and cultured according to the standard DSMZ protocols in DSMZ52 medium. Only 1 mM cysteine was used as a reducing agent. The culture was incubated in serum flasks at 35 °C without shaking. For bioreactor experiments, MgSO_4_ was omitted from the medium and replaced with an equimolar concentration of MgCl_2_·6H_2_O, while the cysteine concentration was increased from 1 to 4 mM. In addition, we adapted the concentrations of ethanol and acetic acid to obtain the desired molar ratio of ethanol/acetic acid (10:1, later 3:1) and organic loading rate (OLR) (Table 1). For the batch experiments, two types of syngas fermentation effluent were tested. The first effluent type was obtained from the second stage of a two-stage syngas fermenter operated as described by Richter et al. [31]. The influent of the syngas fermenter consisted of 2× concentrated P7 medium (Additional file 1, [32]). The second effluent type was collected from a single-stage syngas fermenter operated with mineral medium, which was adapted from Mock et al. [33] (Additional file 1). We used the second effluent type for our bioreactor experiments. The collected effluent was kept frozen (−20 °C) until use. Next, the effluent was filtered with a 0.2-µm vacuum filter to obtain a sterile influent for the *C. kluyveri* experiments. The concentrations of ethanol and acetic acid were determined in the filtered substrate and ethanol was supplemented when needed to obtain the desired concentration (Table 1). Trace elements, vitamins, selenite–tungstate (as for DSMZ52 medium), and cysteine were added after filtration. We omitted yeast extract from the bioreactor experiments.

**Table 1:** Theoretical composition of the substrate (ethanol and acetic acid) during the operating period for the bioreactor with pertraction (BP) and without pertraction (BNP)

Day (Phase)	OLR BP (g COD L^−1^ day^−1^; mM-C day^−1^)	OLR BNP (g COD L^−1^ day^−1^; mM-C day^−1^)	HRT (d)	Ethanol/acetic acid ratio
1–18 (I)	12 (257)	12 (257)	2	10
18–28 (II)	6 (129)	6 (129)	4	10
28–37 (III)	6 (129)	10 (215)	2	10
37–48 (IV)	10 (215)	10 (215)	2	10
48–74 (V)	15 (322)	15 (322)	2	10
74–85 (VI)	15 (340)	15 (340)	2.3	3
86–95 (VII)^a^	15 (340)	15 (340)	2.3	3

Ethanol and acetic acid have a COD content of 96 and 64 g COD mol^−1^, respectively

*HRT* hydraulic residence time, *COD* chemical oxygen demand, *OLR* organic loading rate

^a^Real syngas fermentation effluent was used

### Batch-growth experiment with syngas fermentation effluent

Growth and production of *C. kluyveri* were tested in eight different media with and without additions of various chemicals to evaluate the use of syngas fermentation effluent as growth medium for a *C. kluyveri* bioreactor (Table 2). The eight different media used in the batch test were as follows: (i) DSMZ52 *C. kluyveri* medium (DSMZ); (ii) P7 syngas fermentation effluent with only bicarbonate added (SGP−); (iii) P7 syngas fermentation effluent with trace elements, vitamins, selenite–tungstate, and bicarbonate added (SGPT−); (iv) P7 syngas fermentation effluent with trace elements, vitamins, selenite–tungstate, and bicarbonate, as well as yeast extract added (SGPT+); (v) Mock syngas fermentation effluent with only bicarbonate added (SGM−); (vi) Mock syngas fermentation effluent with trace elements, vitamins, selenite–tungstate, and bicarbonate added (SGMT−); (vii) 2× concentrated P7 medium with only bicarbonate added (P−); and (viii) 2× concentrated Mock medium with only bicarbonate added (M−). Trace elements, vitamins, and selenite–tungstate solutions were prepared and added according to the DSMZ52 medium recipe (Additional file 1).

**Table 2:** Composition of the media tested in the batch-growth experiments

Condition	Ethanol (mM)	Acetic acid (mM)	Vitamins	Trace elements	Selenite–tungstate	Yeast extract	Bicarbonate
DSMZ	343	101	Y	Y	Y	Y	Y
SGP−	315	144	N	N	N	N	Y
SGPT−	315	144	Y	Y	Y	N	Y
SGPT+	315	144	Y	Y	Y	Y	Y
SGM−	343	101	N	N	N	N	Y
SGMT-	343	101	Y	Y	Y	N	Y
P−	343	101	Y	Y	Y	N	Y
M−	343	101	N^a^	N^a^	N^a^	N	Y

The test was carried out as three separate experiments, each time with DSMZ52 medium (DSMZ) as the control. Each medium contained the same COD concentration. Additions were made based on the DSMZ52 medium. Ethanol and acetic acid concentrations depicted here are the theoretical concentration

*N* not added, *Y* added

^a^The vitamins and trace elements for the 2× Mock medium were used

All media contained 1 mM cysteine as a reducing agent. The combined chemical oxygen demand (COD) from ethanol and acetic acid was the same for each medium. The 2× P7 medium was prepared using the mineral solution of the P7 medium, with additions according to the DSMZ52 medium and the COD and ethanol/acetic acid ratio of the DSMZ52 medium. The 2× Mock medium was prepared with only ethanol and acetic acid added before the transfer of *C. kluyveri* (Additional file 1). The experiment was carried out in three separate batches, each time with the DSMZ52 medium as the control. An active *C. kluyveri* culture growing in DSMZ52 medium was used as inoculum, and transferred in each specific medium for two growth cycles to dilute the original DSMZ52 medium and to allow adaptation of the culture. A triplicate bottle test was subsequently carried out. Incubation (without shaking) of the triplicate bottle test took place at 30 °C, which was the same temperature used for bioreactor operation. The pH of the media was not controlled during the experiment (Additional file 2).

### Bioreactor setup for continuous experiments

Two BioFlo310 Benchtop fermenters (New Brunswick Scientific Co, CT, USA), with a 2.5-L vessel (operating volume of 1.5 L), were used for the bioreactor experiments. The headplate of each bioreactor was equipped with a sampling port; gas sparger; exhaust condenser; mixing system (200 rpm); a pH probe (Mettler Toledo 405-DPAS-SC, Mettler Toledo, OH, USA); an inlet for influent, acid, and base; an outlet for both recirculation and effluent; and an inlet for the recirculated broth. The temperature was controlled at 30 °C using the built-in water jacket. Each bioreactor system included an external recirculation pump (Masterflex, IL, USA). Bioreactor broth was recirculated at 120 mL min^−1^ over a hollow-fiber membrane (Cell guard, Minikros Sampler, 20 cm, pore size 0.2 µm, PES, Spectrumlabs, CA, USA) to retain all cells in the bioreactor. The cell-free medium that we obtained from the hollow-fiber unit was pumped out as effluent at a rate of 750 mL day^−1^. The same volume of influent medium was pumped in daily, resulting in an HRT of ~2 days. The bioreactors were continuously sparged with anaerobic gas, consisting of 20 mL min^−1^ N_2_ and 5 mL min^−1^ CO_2_, at a flow rate of ~25 mL min^−1^. The flow rates of the two gases were manually controlled using flow meters (65-mm Correlated Flowmeter with valve, aluminum, Cole-Parmer, IL, USA). Both bioreactors were initially controlled at pH 7, using 2 M KOH and 2 M HCl.

### Pertraction system

**Fig. 2:**
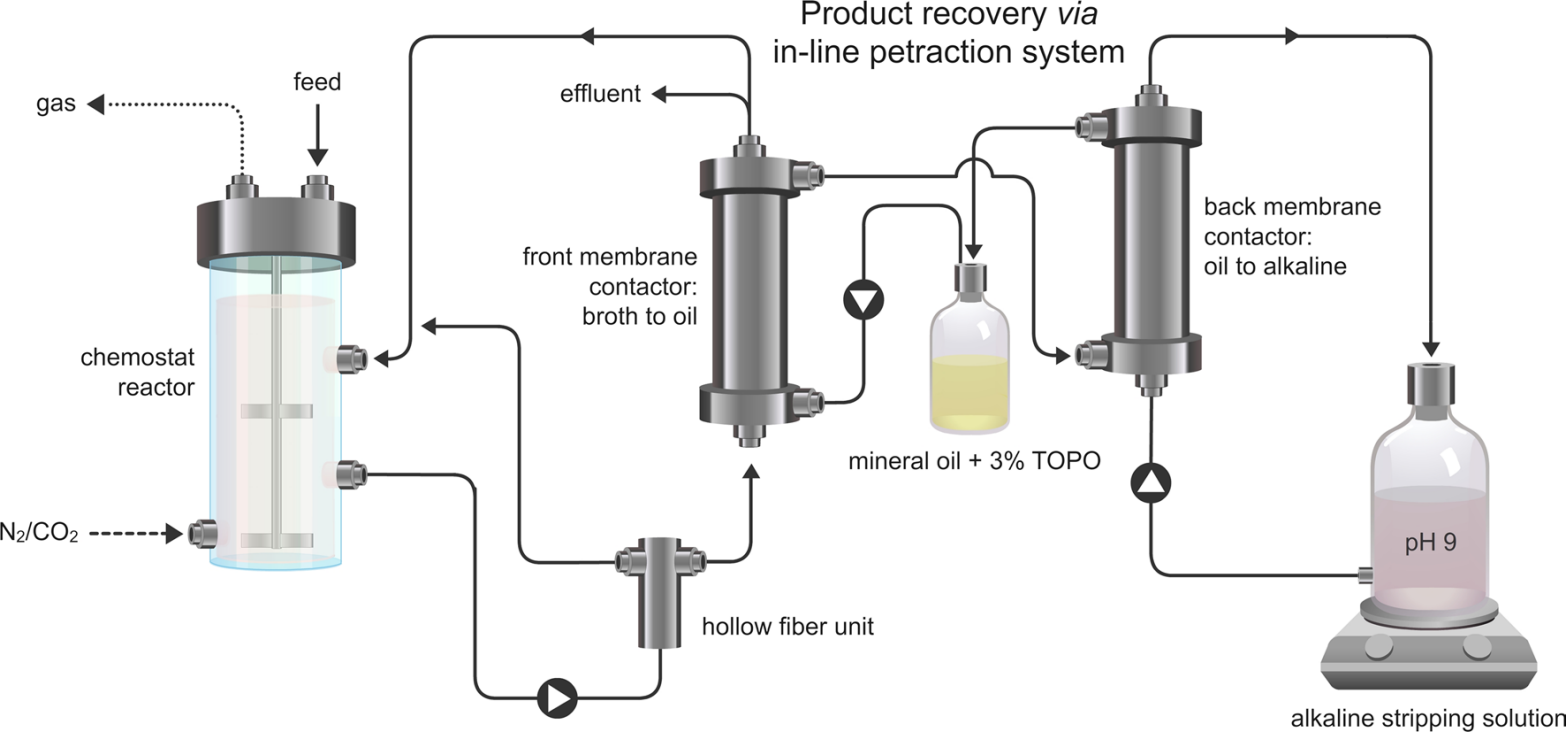
Bioreactor setup for fermentation with in-line product extraction (pertraction). The bioreactor broth is sent through a hollow-fiber unit (cell guard) to obtain a cell-free effluent and broth for the pertraction system

One of the two bioreactors was equipped with a membrane-based liquid–liquid extraction system (pertraction), consisting of a forward and backward extraction module (Fig. 2). We refer to this bioreactor as the bioreactor with pertraction (BP). The other bioreactor was referred to as the bioreactor with no pertraction (BNP). The pertraction system was connected and started on day 11 of the bioreactor experiment. This system extracts carboxylic acids into a solvent, based on their hydrophobicity, and subsequently into an alkaline stripping solution, based on a pH gradient between the bioreactor and the stripping solution [18]. The cell-free filtrate leaving the hollow-fiber unit (cell guard) was pumped at 90 mL min^−1^ into the shell side of the forward extraction module (2.5 × 8 Liqui-Cel Membrane Contactor, Membrana, Germany). There it contacted the hydrophobic solvent, which consisted of mineral oil containing 30 g L^−1^ tri-*n*-octylphosphine oxide (TOPO; Sigma Aldrich, MO, USA). The mineral oil was recirculated on the tube side between the two extraction modules at a flow rate of 7.5 mL min^−1^. In the backward module, the mineral oil contacted the stripping solution, which was recirculated at 75 mL min^−1^. The stripping solution consisted of 300 mM Na_2_B_4_O_7_, which was controlled at pH 9.0 using 5 M NaOH. The stripping solution was regularly refreshed to keep the total carboxylate concentration below 120 mM. We calculated the product formation from the accumulation of product in the stripping solution between two sampling times and the volumetric production rate based on reactor broth concentrations and hydraulic residence time.

### Bioreactor startup and operation

The bioreactors were filled with medium, autoclaved, and sparged with sterile N_2_/CO_2_ at a flow rate of 25 mL min^−1^ for 24 h. Liquid samples were taken before inoculation. Each bioreactor was inoculated at a 7.5% (v/v) concentration using serum flask-cultures of *C. kluyveri*. In the bioreactor experiments described next, continuous mode was started after 12 h in batch mode, at an OLR of 12 g COD L^−1^ day^−1^. In initial runs, continuous-mode operation was started when the optical density (OD) was above 1, or, in a later run this was done as soon as the OD was higher than 0.3. The OLR applied upon transitioning to continuous mode was 2 g COD L^−1^ day^−1^ in the initial runs, and was later increased to 8 g COD L^−1^ day^−1^. The faster transition and increased OLR were chosen because the culture had shown sporulation behavior (visual observation with a phase-contrast microscope, Nikon Labophot, Nikon, NY, USA). The operating period consisted of seven different operating phases, which were separated based on the OLR, the ethanol/acetic acid ratio, or the synthetic vs. real syngas fermentation effluent (Table 1). After two initial phases during which the flow rate was halved (Phase I and II), the OLR was gradually increased during the remaining operating period (Phases III–VII). Liquid samples were taken at least four times per week. Gas flow rate and composition, effluent volume, and volume of dosed acid and base were also measured during sampling. Possible losses of ethanol or carboxylic acids via the exhaust gas were not quantified, although these could have taken place despite the use of an exhaust condenser [34]. In the figures and tables, we show most of the results as mM product. Specificities were calculated as weighted averages of the ratio of the carbon (mM-C) in the product of interest and the carbon (mM-C) in *n*-butyric acid, *n*-caproic acid, and *n*-caprylic acid combined, which were measured during at least three time points during steady state. Carbon conversion efficiencies were calculated as weighted averages based on the input carbon (mM-C ethanol and acetic acid) and the carbon for products in both the effluent and extraction solution (mM-C butyric acid, *n*-caproic acid, and *n*-caprylic acid), which were measured during at least three time points during steady state.

Four technical problems decreased the efficiency of the pertraction system at certain time points: (i) mineral oil leakages took place on day 20 and 38; (ii) the actual mineral oil recirculation rate was lower than the set-point from day 30 to 76; (iii) bioreactor broth was lost due to a tubing breakage in the pump line on day 87 (first incident) and 95 (second incident); and (iv) a short circuit in the broth flow was identified and remedied on day 89. Due to this short circuit, bioreactor broth containing biomass was siphoned into the cell-free broth feeding the effluent line. Both bioreactors were discontinued after the second incident involving bioreactor broth loss on day 95.

### Analytical procedures

The OD was measured at 600 nm using a spectrophotometer (Spectronic 1201, Milton Roy, NJ, USA; quartz cuvette, Starna Cells, CA, USA). Phosphate buffer (50 mM, pH 6.8) was used to dilute the samples, if necessary. For the batch experiment, 200 µL broth was analyzed using a 96-well plate in a plate reader (BioTek Synergy 4, BioTek, VT, USA). The reading of the pH probes in the bioreactors was controlled at least four times per week by measuring the pH with an external pH probe, calibrated using buffers at pH 4, 7, and 10 (Orion Star A329, Thermo Fischer Scientific, MA, USA).

Gas samples were analyzed on a gas chromatograph (GOW-MAC Instrument Co., PA, USA), which was equipped with a column (1.8 m; 80/100 Hayesep Q packing material; Supelco, PA, USA) at 25 °C, using helium as the carrier gas. The ratio of the integrated peak areas for N_2_ and CO_2_ was determined using PeakSimple software v4.44 (Schemback, Germany). Ethanol and acetic acid concentrations were determined using a HPLC system (Shimadzu Prominence HPLC system, Columbia, MD, USA), which was equipped with the following components: the SIL-20AC HT autosampler, RID-10A refractive index detector, CTO-20AC column oven, and the CBM-20A communications bus module. Compounds were separated in an Aminex HPX-87H analytical column at 65 °C (Bio-Rad, CA, USA), using sulfuric acid (5 mM, flow rate 0.6 mL min^−1^) as the mobile phase. The Shimadzu LabSolutions data analysis software was used to integrate ethanol and acetic acid peaks. Concentrations of carboxylic acids (C4–C8) were measured using a gas chromatograph (HP5890, Hewlett Packard, USA), which was equipped with a 7683 auto-injector and flame ionization detector. Columns [capillary GC column (Nukol); 15 m × 0.53 mm internal diameter (Supelco)] were purchased from Sigma Aldrich, Inc. (USA). The flow rates of hydrogen, air, and helium were 21.4, 350, and 35 mL min^−1^, respectively. The temperature was first set to 70 °C for 2 min, and then ramped up to a final temperature of 200 °C at 12 °C min^−1^, where the temperature was held for 2 min. The injection port temperature and detector temperature were 200 and 275 °C, respectively.

### Microbiome analysis

Biomass samples were collected from both BP and BNP at two time points: day 37 and 45. Approximately 2 mL of bioreactor broth was centrifuged at 13,000 rpm (Eppendorf Centrifuge 5415D, Eppendorf, Hauppauge, NY, USA) and the supernatant was discarded. Samples were stored at −20 °C until further processing. Genomic DNA was extracted from bioreactor samples using the MO BIO PowerSoil kit (MO BIO Laboratories Inc., Carlsbad, CA, USA). Modifications to the manufacturer’s protocol were described previously [35]. In brief, custom bead tubes with 0.1-mm and 0.5-mm diameter silica/zirconia beads were assembled in a sterile laminar flow hood. Cell lysis for DNA isolation was performed by bead-beating at 3550 oscillations per min for 45 s. To profile the microbial community within each sample, DNA samples were amplified for the V4 region of the 16S rRNA gene. The PCR amplification protocol was described previously [35]. Purified PCR amplicons were pooled to 4 ng per sample, and submitted for Illumina MiSeq sequencing (Cornell Genomics Facility, Cornell University Institute of Biotechnology, Ithaca, NY). We used QIITA (qiita.microbio.me) to quality filter and demultiplex raw sequences. Sequences were separated into groups (or operational taxonomic units, OTUs) of similar sequences (97% similarity) using the sortmerna method. OTUs were referenced against the Greengenes v13.8 database [36]. Singletons (rare OTUs) were removed to filter out OTUs with one count across all samples. Bacterial abundance profile was calculated in MacQIIME v1.9.1 [37].

## Results and discussion

### Trace elements and/or vitamins must be added to syngas fermentation effluent to grow *C. kluyveri*

**Fig. 3:**
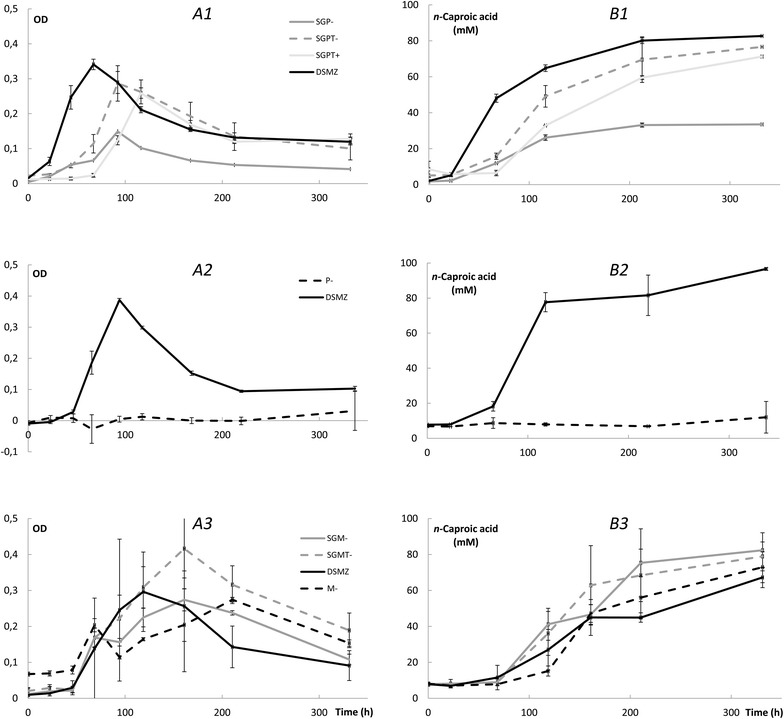
Three separate batch experiments (1, 2, and 3) were carried out. **a** Growth (as OD) was highest for the standard DSMZ52 (DMSZ) medium and the Mock medium syngas fermentation effluent with added growth factors, but no yeast extract. **b** Production of *n*-caproic acid was the highest in the standard DSMZ52 medium, but comparable results were obtained with syngas fermentation effluent with additions. Data represent the mean (*n* = 3), *error bars* indicate the standard deviation

Yeast extract is often added to enhance growth and production rates of *C. kluyveri* although it is known that it can be substituted by biotin and *p*-aminobenzoic acid [38, 39]. Both batch-growth experiments with (1) original DSMZ52 medium (DSMZ) with yeast extract and (2) Mock syngas fermentation effluent with trace elements, vitamins, and selenite–tungstate added, but without yeast extract (SGMT−) showed the fastest growth, highest OD, and highest concentration of *n*-caproic acid produced after 2 weeks (Fig. 3 A1–B3). In addition, P7 syngas fermentation effluent-based medium with additional trace elements, vitamins, and selenite–tungstate, but without yeast extract (SGPT−), resulted in shorter lag phase than the same medium with yeast extract (SGPT+) (Fig. 3 A1). In all the fermentation broth solutions of the batch-growth experiments, the pH values remained above 6 (Additional file 2). From these results, we concluded that the addition of undefined yeast extract is not necessary to sustain growth of *C. kluyveri* when coupling syngas fermentation and chain elongation. However, the use of raw syngas fermentation effluent is also not possible because growth in this condition was retarded for the P7-based effluent (SGP−) (Fig. 3 A1, B1). Certain trace elements or vitamins are missing in the raw syngas fermentation effluent. This effluent contains cell material from dead cells or excretion products from living cells during syngas fermentation and the unused trace elements, vitamins, and nutrients that were added to the original substrate of the syngas fermentation system. Yeast extract is typically not added to syngas fermentation with the goal to boost the production of ethanol, and therefore some trace element and/or vitamin is likely missing. Before developing an industrial-scale system, syngas fermentation effluent should be fully characterized. An economic analysis is then needed to ascertain whether the introduction of certain trace elements or vitamins vs. the addition of yeast extract is more economically attractive [40].

### *Clostridium kluyveri* can produce *n*-caprylic acid (C8)

In seven out of eight medium conditions during the batch-growth experiments (all except P−), *n*-caprylic acid was detected, with the highest concentrations occurring with Mock syngas fermentation effluent (2.19 ± 0.34 mM after 14 days) (Additional file 3). *n*-Caprylic acid production has been reported for reactor microbiomes, including those with a high abundance of *C. kluyveri* [18, 27, 41]. However, this is the first time that production of *n*-caprylic acid is reported for a pure culture of *C. kluyveri*. Chain elongation takes place through the reverse β-oxidation pathway, which is a cyclic process (Fig. 1). In the first step, two acetyl-CoA molecules are combined to form butyryl-CoA, which may be reduced to *n*-butyric acid. Alternatively, butyryl-CoA may enter a second round of chain elongation where it is coupled to another acetyl-CoA forming hexanoyl-CoA, which may then be reduced to *n*-caproic acid. Most likely, *C. kluyveri* has the capacity to produce *n*-caprylic acid through a similar cycle, coupling acetyl-CoA to hexanoyl-CoA under more reduced conditions [27, 42]. Carboxylic acids generally become more toxic with increasing chain length. This toxicity is a possible explanation for the absence of *n*-caprylic acid detection in previous studies. The other explanation is that *n*-caprylic acid was simply not analyzed.

### Continuous-mode operation with *C. kluyveri* requires a specific startup procedure

Initial attempts to start the bioreactors with synthetic medium were not successful due to two key reasons: (1) contamination; and (2) sporulation. First, sulfate-reducing bacteria (SRB) contaminated the culture despite our axenic culturing methods, resulting in an H_2_S-like odor. The SRB reduce sulfate, using H_2_ or acetic acid as electron donor [43]. Hydrogen gas is produced by *C. kluyveri* during chain elongation, while acetic acid (electron acceptor) and sulfate (sulfur source) are components of the DSMZ52 medium used for this study. To prevent contamination, sulfate was replaced by cysteine as the sulfur source. We postulated that this would not affect *C. kluyveri* because they are endowed with cysteine degradation genes [44]. As anticipated, growth of the culture was not affected by the change from sulfate to cysteine as the sulfur source. SRB contamination was successfully avoided in the subsequent bioreactor runs.

Second, we visually observed sporulation of the culture with a phase-contrast microscope (Additional file 4). We hypothesized that sporulation was triggered by a lack of carbon source or nutrients at the end of the exponential growth phase during the batch-wise startup or just after the switch to the continuous-mode operation [45]. However, a faster transition to continuous mode or higher loading rates did not avoid sporulation. The use of the hollow-fiber unit to retain cells in the bioreactor makes the startup in batch mode unnecessary for our continuous-mode operation. Therefore, the bioreactors were subsequently started in continuous mode directly at a loading rate of 12 g COD L^−1^ day^−1^. However, using this operating strategy, the culture still sporulated. In fact, sporulation was initiated even faster compared to batch-mode operation (day 3 instead of day 5). Fortunately, the spores also germinated again within 2 days, forming thinner *C. kluyveri* cells compared to the cells from the initial culture (Additional file 4). The culture regained its original appearance during the operating period, leading to successful runs. Since we stopped the earlier runs immediately after we had observed sporulation, we do not know whether germination would or would not have occurred then. The sporulation and germination behavior of *C. kluyveri* should be further studied in detail to understand the mechanisms, and possible impacts on long-term reactor operation.

**Fig. 4:**
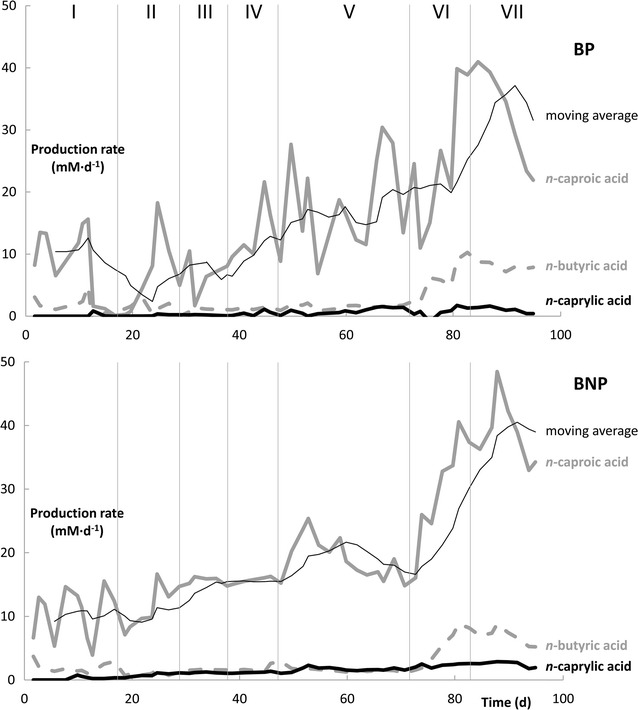
Production rates of *n*-butyric acid (*gray dotted line*), *n*-caproic acid (*gray line*), and *n*-caprylic acid (*black line*) during continuous-mode operation of the bioreactor with pertraction (BP, *top*, operating pH 6) and without pertraction (BNP, *bottom*, operating pH 7). The moving average (*n* = 7) for the *n*-caproic acid concentration is provided. The seven operating phases (Table 2) are marked with *vertical lines*

Both BP and BNP showed similar behavior in the 10 days after startup in continuous mode and before the pertraction was initiated for BP (Fig. 4). During Phase I of the operating period, the optical density increased within 3 days, dropped when sporulation occurred (Additional file 4), and increased again when the cells germinated (Additional file 5). Production of *n*-butyric acid and *n*-caproic acid followed the growth pattern, resulting in an *n*-caproic acid concentration of 33 mM for BP and 31 mM for BNP on day 3 after which a drop in production occurred due to sporulation between day 3 and 6 (Additional file 6). The production for BNP then followed a cyclic pattern, with either peaking substrate concentrations (ethanol and acetic acid in Additional files 6, 7) or product concentrations (*n*-butyric and *n*-caproic acid in Additional file 6). Metabolic oscillations coupled to sporulation have been previously reported for solventogenic Clostridia due to a periodic shift between acidogenic (primary) and solventogenic (secondary) metabolisms [46–49]. The cyclic behavior disappeared when the loading rate was decreased to 6 g COD L^−1^ day^−1^ on day 17 during Phase II (Fig. 4).

Pure-culture fermentations are often executed in batch-mode operation rather than in continuous-mode operation. Our results suggest that continuous-mode operation would be more advantageous. The batch-growth experiment and Phase VI of the operating period for the continuous-mode operation can be compared because similar ratios of ethanol and acetic acid were used (3:1). In the continuous mode, the *n*-caproic acid production rates, specificities, and conversion efficiencies were always higher (Table 3). In a continuous mode and with a completely mixed system, the ethanol concentration will be low due to substrate removal when the system is not overfed, which prevents ethanol inhibition [23]. In addition, the continuous removal of product with the effluent will reduce the accumulation of MCCAs, circumventing the product inhibition that is anticipated at the end of the operating period of a batch-mode operation. The faster sporulation behavior of our culture during continuous-mode operation did not negatively affect process outcomes in the long term.

**Table 3:** Overview of critical performance parameters for the bioreactor with pertraction (BP; pH 6) and the bioreactor without pertraction (BNP; pH 7) for the different operating phases as defined in Table 1 (average ± stdev)

	Phase	OLR (g COD L^−1^ day^−1^)	OLR (mM-C day^−1^)	*n*-Butyric acid (mM day^−1^)	*n*-Caproic acid (mM day^−1^)	*n*-Caprylic acid (mM day^−1^)	Specificity C6 (%)	Specificity C8 (%)	Carbon conversion efficiency (%)	Extr. C6 (%)	Extr. C8 (%)
BP	II–III	7.0 ± 0.4	151.9 ± 31.4	0.9 ± 0.7	8.4 ± 8.4	0.2 ± 0.1	88.5 ± 10.1	3.7 ± 2.9	38.9 ± 17.4	80.4 ± 10.5	100.0 ± 0.0
	IV	10.9 ± 0.4	255.2 ± 7.4	1.3 ± 0.2	16.2 ± 5.4	0.6 ± 0.4	90.9 ± 2.0	3.9 ± 2.2	41.9 ± 2.5	64.8 ± 13.7	94.4 ± 6.6
	V	14.2 ± 1.0	298.3 ± 9.6	1.7 ± 0.4	19.1 ± 6.6	1.0 ± 0.4	87.7 ± 3.9	6.4 ± 3.0	38.5 ± 12.6	37.8 ± 27.2	65.0 ± 26.5
	VI	16.3 ± 0.4	370.0 ± 9.7	9.4 ± 0.7	39.9 ± 0.9	1.4 ± 0.2	83.0 ± 1.0	4.0 ± 0.4	79.9 ± 2.0	42.7 ± 6.9	77.0 ± 4.5
	VII	10.7 ± 3.3	244.6 ± 74.4	7.8 ± 0.11	23.1 ± 4.7	0.47 ± 0.3	79.5 ± 1.9	1.9 ± 1.6	76.4 ± 19.5	70.0 ± 40	101 ± 2.7
BNP	II	6.9 ± 0.2	122.5 ± 31.7	1.0 ± 0.3	11.1 ± 2.7	0.8 ± 0.2	86.5 ± 0.5	8.1 ± 0.2	65.9 ± 17.4		
	III–IV	10.9 ± 0.4	233.0 ± 7.9	1.7 ± 0.3	15.7 ± 0.5	1.2 ± 0.1	85.5 ± 1.2	8.5 ± 0.4	47.5 ± 2.5		
	V	11.3 ± 1.2	241.0 ± 28.4	1.5 ± 0.2	18.6 ± 2.9	1.8 ± 0.3	84.7 ± 1.5	10.8 ± 1.2	54.7 ± 6.5		
	VI	13.0 ± 0.03	296.5 ± 0.7	7.8 ± 0.7	37.6 ± 1.6	2.6 ± 0.01	81.4 ± 0.5	7.4 ± 0.3	93.6 ± 4.4		
	VII	12.3 ± 1.2	279.7 ± 27	5.7 ± 0.7	35.3 ± 2.7	2.14 ± 0.4	84.2 ± 1.0	6.7 ± 0.7	90.1 ± 0.7		

*COD* chemical oxygen demand, *C6 n*-caproic acid, *C8 n*-caprylic acid, *Extr.* extraction efficiency

Production rates for the three products (*n*-butyric, *n*-caproic and *n*-caprylic acid) are shown. Weighted averages were taken for the steady state periods (starting 3 HRTs after a change in condition), and for *n* ≥ 3

### The mildly acidic pH value of 5.5 was not favorable for *C. kluyveri*

The pH for BP was decreased from 7.0 to 5.5 in one step on day 11 to immediately ensure an efficient pertraction rate of the undissociated carboxylic acid products. All *n*-caproic acid, which was present in the bioreactor broth before day 11, was quickly extracted after this pH decrease (Additional file 6), but the production of MCCAs ceased as well (Fig. 4) and the OD decreased (Additional file 5). In early studies with *C. kluyveri*, the pH range that allowed growth was found to be between 6.0 and 7.5, with a pH optimum of 6.4 [26, 28]. A recent isolate of *C. kluyveri* from bovine rumen, however, was able to grow and produce *n*-caproic acid at a pH as low as 4.8 [50]. In addition, a co-culture of *C. autoethanogenum* and *C. kluyveri* was functional at a pH of 5.5, albeit at a relatively low *n*-caproic acid production rate [51]. Open-culture bioreactors with pertraction have been operated at a pH of 5.5 without noticeable challenges, while *C. kluyveri* became enriched with increasing *n*-caproic acid production rates [18, 21].

Bioreactor studies without extraction operate usually at a neutral pH and with higher dilution rates to prevent product toxicity. *C. kluyveri* was found to be abundant in these microbiomes [27]. The toxicity of the undissociated MCCAs at mildly acidic conditions is one main reason for the growth inhibition of *C. kluyveri* (and other bacteria) in microbiomes [18, 27]. However, here we found that the other main reason is the pH value of 5.5 directly as the inhibitor for the growth of *C. kluyveri*, because all MCCAs were extracted from the broth. In future experiments, a more gradual pH decrease from 7 to 5.5 should be tested as an operating strategy. Alternatively, a newly isolated and acid-tolerant chain-elongating strain, or the acid-tolerant *C. kluyveri* strain as described by Weimer et al. [50], could be considered as a more ideal biocatalyst for pure-culture bioprocessing. To restart production in BP, we increased the pH from 5.5 to 6.0 on day 20 of the operating period after which additional inoculum was spiked into the bioreactor. Production of *n*-caproic acid resumed, but during Phase II the production rates and carbon conversion efficiencies remained lower for BP at a pH of 6 than for BNP at a pH of 7 (Additional file 8).

### Pertraction at a pH of 6 was not optimal

The efficiency of the pertraction system in BP was anticipated to be considerably lower at a pH value of 6.0 compared to 5.5. At a pH of 6.0, only 6.6% of the *n*-caproic acid is present in the undissociated form, while this is 18.3% at a pH of 5.5. Because the undissociated acids are transferring from the fermentation broth into the mineral oil through the membrane of the forward module, the pertraction rates were positively correlated to the concentration of undissociated acids at the membrane boundary [52]. At a loading rate of 10 g COD L^−1^ day^−1^ (Phase IV), the average efficiency for *n*-caproic acid extraction was only 65% (Table 3). With open cultures, a removal efficiency of greater than 95% for *n*-caproic acid has been achieved with a similar pertraction system at mildly acidic pH values of 5.5 [53]. During Phase VI, the concentration of undissociated *n*-caproic acid was 3.5 ± 0.4 mM in BP (pH 6) due to the relatively inefficient extraction. This is within the concentration range typically reported to be toxic for pure or open cultures, depending on the concentration of other undissociated carboxylic acids (0.66–7.5 mM; [50, 51, 53]). For BNP (pH 7), the undissociated *n*-caproic acid concentration was 0.54 ± 0.07 mM, which is considerably lower and outside of the inhibiting concentration. At mildly acidic conditions, it is, therefore, essential to operate an optimal pertraction system or other extraction technology to prevent product toxicity.

### We observed similarities and differences between extraction at a pH of 6 (BP) and without extraction at a pH of 7 (BNP)

During Phases III, IV, and V, the OLR was gradually increased (Tables 2, 3). This increase was slower in BP following the decrease in the pH value. For BP, the OLR of 10 g COD L^−1^ day^−1^ resulted in *n*-caproic acid production rates of 16.2 ± 5.4 mM day^−1^ (1.9 g L^−1^ day^−1^; 4.2 g COD L^−1^ day^−1^). For BNP, *n*-caproic acid was produced at a similar rate of 15.7 ± 0.5 mM day^−1^ (1.8 g L^−1^ day^−1^; 4.0 g COD L^−1^ day^−1^) (Fig. 4). These production rates with *C. kluyveri* are not only comparable for these two bioreactors but also for open-culture bioprocessing systems at similar OLRs with a complex substrate and extraction [53]. On day 48 of the operating period (Phase V), we increased the OLR to 15 g COD L^−1^ day^−1^; however, after measurement, the actual loading rates were found to be slightly lower (14.2 and 13.0 g COD L^−1^ day^−1^; Table 3). *n*-Caproic acid production rates during the steady state period at this loading rate (days 55–74) were 19.1 ± 6.6 mM day^−1^ (2.21 g L^−1^ day^−1^; 4.9 g COD L^−1^ day^−1^) and 18.6 ± 2.9 mM day^−1^ (2.16 g L^−1^ day^−1^; 4.8 g COD L^−1^ day^−1^) for the BP and BNP, respectively (Table 3). The production rates were, thus, similar for these bioreactors.

*n*-Caproic acid was the main product at an OLR of 10 g COD L^−1^ day^−1^ during Phase IV with a specificity of 90.9 ± 2.0 and 85.5 ± 1.2% in their products for BP and BNP, respectively (Table 3). The other products of chain elongation were *n*-butyric and *n*-caprylic acid. *n*-Caprylic acid specificity was limited to 3.9 ± 2.2% for BP and 8.5 ± 0.4% for BNP (Table 3). Although *n*-caprylic acid is preferentially extracted from BP via pertraction compared to *n*-caproate, we observed a higher *n*-caprylic acid production rate without extraction at a pH of 7 (BNP). We postulate that the higher pH value of 7 compared to 6 reduced the toxicity for undissociated *n*-caprylic acid enough to explain the higher *n*-caprylic acid specificities, but more research is necessary to substantiate this claim. More research is also required to understand the long-term effect of *n*-caprylic acid production on the culture, as well as how metabolism can be steered toward this product. Kucek et al. [22] did achieve a much higher *n*-caprylic acid specificity at a mildly acidic pH value with a microbiome that was supplied with a similar synthetic syngas fermentation effluent with an ethanol/acetic acid ratio of 10:1 (molar basis) similar to our study. However, their microbiota did not contain *C. kluyveri* as a dominant bacterium.

Sequencing revealed a certain degree of contamination in the bioreactors (Additional file 9). The contamination did not affect bioreactor functionality because promising conversion rates were obtained for both bioreactors. Other pure culture processes, such as yeast ethanol fermentation and biopharmaceutical production, typically encounter a certain level of contamination as well (up to 5% relative abundance), which does not affect the production process [54]. Contamination was higher in BP (Additional file 9), which could be due to: (i) the inability to sterilize the extraction modules prior to use; (ii) an accident that could have contaminated the substrate bottle on day 43; and (iii) the occurrence of a short circuit in the system, rendering the bioreactor vessel in contact with the open air via the effluent line. The increase in the complexity from adding product extraction rendered the system more prone to contamination. The majority of the contaminants belonged to an unknown family in the order Burkholderiales (Additional file 9). The 16S rRNA gene from this microbe is 100% identical compared to a microbe that was found within a natural biofilm on stainless steel and PVC [55]. In BP, the relative abundance of *C. kluyveri* increased while the abundance of contaminants decreased from day 37 to 45. This indicates community resilience, and that events causing contamination did not lead to dominance of contaminating species during the operating period of our study.

### The carbon efficiency increased when we lowered the ethanol/acetic acid ratio

During Phase VI, the ethanol/acetic acid ratio in the synthetic feed was decreased from 10:1 to 3:1 to match the ratio in the real syngas fermentation effluent that was available. This resulted in two changes. First, the specificity for *n*-caprylic acid decreased (Table 3), which is similar to the results by Kucek et al. [22]. The specificity for *n*-caproic acid remained relatively stable, however, because proportionally more *n*-butyric acid was produced rather than *n*-caprylic acid. The lower specificity was anticipated as early work already found that a high ethanol/acetic acid ratio is beneficial to obtain longer chain products [29]. Second, the carbon conversion efficiency increased with lower ethanol/acetic acid ratios (Table 3). The increase in efficiency can be partly explained for BNP due to the complete consumption of ethanol after the ratio decrease (Additional file 7). In addition, for BP and BNP, the production rates of all three carboxylic acids increased with an increase in *n*-caproic acid production rates to 39.9 ± 0.9 mM day^−1^ (4.6 g L^−1^ day^−1^; 10.2 g COD L^−1^ day^−1^) and 37.6 ± 1.6 mM day^−1^ (4.4 g L^−1^ day^−1^; 9.7 g COD L^−1^ day^−1^), respectively. At this point, we can only speculate about why this occurred. When the consumed ethanol/acetic acid ratio is higher, more ATP is produced, and as a result more biomass is grown in the bioreactor [23, 29, 56]. Within the reverse β oxidation pathway, ATP is formed via both substrate-level phosphorylation and transport-coupled phosphorylation [25, 42]. During a period of higher ethanol availability, the anaerobic oxidation of ethanol, which is coupled to chain elongation, would provide more energy through substrate-level phosphorylation. Lowering the ethanol/acetic acid ratio would shift more ATP production toward transport-level phosphorylation. We speculate that chain elongation becomes more efficient as a result due to lower biomass production. The higher substrate conversion efficiency may then have been a mechanism to compensate for the decreased ATP production [25]. However, further research, including thermodynamic and metabolic modeling, is needed to substantiate this hypothesis.

### Chain elongation can be coupled to syngas fermentation with a pure culture

Real syngas fermentation effluent was used as the substrate for both bioreactors starting on day 86 (Phase VII). *n*-Caproic acid was produced at a rate of 35.3 ± 2.7 mM day^−1^ (4.1 g L^−1^ day^−1^; 9.0 g COD L^−1^ day^−1^) for BNP, corresponding to a specificity of 84.2 ± 1.0% (Table 3). Influent carbon was converted to products (*n*-butyric acid, *n*-caproic acid, and *n*-caprylic acid) at an efficiency of 90.1 ± 0.7% (Table 3). We did not observe a decrease in production rate or conversion efficiency when we switched from synthetic to real syngas fermentation effluent (without yeast extract, but with trace elements, vitamins, selenite–tungstate, and cysteine). Therefore, a two-step process for the conversion of syngas fermentation effluent to MCCAs was successfully implemented. No conclusions can be drawn for BP, because steady state had not been achieved when the experiment was terminated on day 95 due to an accident.

Diender et al. and Richter et al. have recently shown that a direct conversion of syngas to *n*-caproic acid with a co-culture of a carboxydotrophic bacterium and *C. kluyveri* is also possible [51, 57]. However, they found that a pH discrepancy exists between the two members in the co-culture, and that *n*-caproic acid was further reduced to *n*-hexanol, resulting in a low specificity for either product. Therefore, a two-stage system would allow better control of process conditions by providing different pH values and temperatures for the two bioprocesses. In addition, the syngas fermentation in the first stage can be operated specifically to achieve the optimum ethanol/acetic acid ratio for further chain elongation in the second stage. It should also be noted that the first stage (syngas fermentation) has already undergone considerable optimization and that full-scale plants are being built. From a practical point of view, it may be easier to build another stage for chain elongation after the existing syngas fermentation stage.

## Conclusions

This is the first long-term continuous bioreactor study using a pure culture of *C. kluyveri* as the biocatalyst for chain elongation. We observed *n*-caprylic acid production by *C. kluyveri* for the first time. Coupling the syngas platform and carboxylate platform was feasible without the addition of yeast extract, and instead with the addition of trace elements and vitamins. We observed sporulation events of the *C. kluyveri* culture during startup periods, but these events did not appear to negatively affect the long-term operating conditions. When in-line *n*-caproic acid extraction via pertraction is desired, the mildly acidic condition (pH of 5.5), which is necessary for an efficient pertraction system, inhibited *C. kluyveri* directly and not only via the increased toxicity of the undissociated *n*-caproic acid or undissociated *n*-caprylic acid. Therefore, a different chain-elongating bacterium with a lower pH optimum than 6–7 should be isolated.

Obtaining a high-rate chain elongation process will be crucial for a successful coupling of syngas fermentation and chain elongation. If methanogenesis and other competing pathways can successfully be inhibited in open culture fermentations, this fermentation strategy would be more advantageous than a pure culture process due to the simplification of the bioprocess without the requirement for axenic conditions. The study presented here was, however, a first proof-of-concept that a pure culture of *C. kluyveri* can act as the catalyst in this bioprocess. Follow-up studies should focus on the influence of pH on the metabolism of *C. kluyveri* in continuous processes or the selection of a different acid-tolerant biocatalyst, as well as the influence of product extraction with an optimized design at high loading rates.

## Additional files

**Additional file 1.** Media used for syngas fermentation; Media compositions.

**Additional file 2.** Batch growth experiment: pH profile; Figure S1 with heading and explanation.

**Additional file 3.** *n*-Caprylic acid concentrations during batch experiments; Table S1 with heading and explanation.

**Additional file 4.** Visual observation of the sporulation behavior of *C. kluyveri*; Figure S2 with heading and explanation.

**Additional file 5.** Optical density; Figure S3 with heading and explanation.

**Additional file 6.** Word document (.docx); Concentrations of carboxylic acids in the bioreactor broth; Figure S4 and S5 with headings and explanations.

**Additional file 7.** Ethanol concentrations in the bioreactors; Figure S6 with heading and explanation.

**Additional file 8.** Conversion efficiency; Figure S7 with heading and explanation.

**Additional file 9.** Sequencing data; Figure S8 with heading and explanation.

## Abbreviations

COD: chemical oxygen demand
MCCA: medium-chain carboxylic acid
OD: optical density at 600 nm
OLR: organic loading rate
SCCA: short-chain carboxylic acid
SRB: sulfate-reducing bacteria
