# Supplementary material for: Upgrading syngas fermentation effluent using Clostridium kluyveri in a continuous fermentation
Source: Biotechnol Biofuels. 2017 Mar 29;10:83. doi: 10.1186/s13068-017-0764-6 (PMC5372331; doi:10.1186/s13068-017-0764-6)
Supplement: Supplementary file 1 — Additional file 1. Media used for syngas fermentation; Media compositions. [file 13068_2017_764_MOESM1_ESM.docx]

## Media used for syngas fermentation

- 1. ***P7 medium***

A 2x concentrated, adapted P7 medium was used. Besides the minerals, trace elements, and vitamins below, 1 mM cysteine and 1 mM Na_2_S were added to the medium.

Mineral solution for 2x P7 medium (60 mL·L^‑1^):

Sodium chloride 80 g·L^‑1^

Ammonium chloride 100 g·L^‑1^

Potassium chloride 10 g·L^‑1^

Potassium phosphate monobasic 10 g·L^‑1^

Magnesium chloride 16 g·L^‑1^

Calcium chloride 4 g·L^‑1^

100x Vitamin solution for 2x P7 medium (20 mL·L^‑1^):

Pyridoxine 0.01 g·L^‑1^

Thiamine 0.005 g·L^‑1^

Riboflavin 0.005 g·L^‑1^

Calcium pantothenate 0.005 g·L^‑1^

Thioctic acid 0.005 g·L^‑1^

Amino benzoic acid 0.005 g·L^‑1^

Nicotinic acid 0.005 g·L^‑1^

Vitamin B12 0.005 g·L^‑1^

Biotin 0.002 g·L^‑1^

Folic acid 0.002 g·L^‑1^

Mercaptoethanesulfonic acid

sodium salt 0.01 g·L^‑1^

100x Trace metals solution for 2x P7 medium (20 mL·L^‑1^):

Nitrilo triacetic acid 2 g·L^‑1^

Manganese chloride 0.833 g·L^‑1^

Ferrous ammonium sulfate 0.8 g·L^‑1^

Cobalt chloride 0.2 g·L^‑1^

Zinc sulfate x 7 H_2_O 0.356 g·L^‑1^

Copper chloride 0.02 g·L^‑1^

Nickel chloride 0.02 g·L^‑1^

Sodium molybdate 0.02 g·L^‑1^

Sodium selenite 0.018 g·L^‑1^

Sodium tungstate x 2 H_2_O 0.022 g·L^‑1^

- 1. **“Mock” medium**

A medium adapted from Mock et al. (2016; see bibliography in main text) was used in a second syngas fermentation experiment. This medium was also a 2x concentrated medium. The second batch of effluent was obtained from this experiment.

Mineral solution (60 mL/L):

Sodium chloride 0 g·L^‑1^ (added separately, 0.24 g·L^‑1^ final concentration in medium)

Ammonium chloride 100 g·L^‑1^

Potassium chloride 5 g·L^‑1^

Potassium phosphate monobasic 27.23 g·L^‑1^

Magnesium chloride x 6 H_2_O 13.33 g·L^‑1^

Calcium chloride 9.8 g·L^‑1^

100x Vitamin solution (20 mL·L^‑1^):

Pyridoxine 0.01 g·L^‑1^

Thiamine 0.05 g·L^‑1^

Riboflavin 0.05 g·L^‑1^

Calcium pantothenate 0.05 g·L^‑1^

Thioctic acid = alpha-lipoic acid 0.05 g·L^‑1^

Amino benzoic acid 0.05 g·L^‑1^

Nicotinic acid 0.05 g·L^‑1^

Vitamin B12 0.05 g·L^‑1^

Biotin 0.02 g·L^‑1^

Folic acid 0.02 g·L^‑1^

Mercaptoethanesulfonic acid

sodium salt 0.01 g·L^‑1^

100x Trace metals solution (20 mL·L^‑1^):

Nitrilo triacetic acid 2 g·L^‑1^

Manganese chloride 0.0252 g·L^‑1^ (200 µM)

Ferrous ammonium sulfate 2.84 g·L^‑1^ (10 mM)

Cobalt chloride 0.0476 g·L^‑1^ (200 µM)

Zinc sulfate x 7 H_2_O 0.2 g·L^‑1^ (700 µM)

Nickel chloride x 6 H_2_O 0.119 g·L^‑1^ (500 µM)

Sodium molybdate 0.0484 g·L^‑1^ (200 µM)

Sodium selenite 0.0346 g·L^‑1^ (200 µM)

Sodium tungstate x 2 H_2_O 0.0660 g·L^‑1^ (200 µM)
