# Supplementary material for: Upgrading syngas fermentation effluent using Clostridium kluyveri in a continuous fermentation
Source: Biotechnol Biofuels. 2017 Mar 29;10:83. doi: 10.1186/s13068-017-0764-6 (PMC5372331; doi:10.1186/s13068-017-0764-6)
Supplement: Supplementary file 2 — Additional file 2. Batch growth experiment: pH profile; Figure S1 with heading and explanation. [file 13068_2017_764_MOESM2_ESM.docx]

## Batch growth experiment: pH profile

Eight different media types (**Table 1** in main text) were used as growth medium for *C. kluyveri*. The pH of the different media did not decrease below 6 (**Figure S1**).

Figure S1 - pH profile of eight media tested for chain elongation. The standard DSMZ 52 medium was used as control each time. SG: syngas fermentation effluent; P: 2x P7 medium; M: 2x Mock medium; T: additions (trace elements, vitamins, selenite-tungstate); -: no yeast extract; +: yeast extract added; DSMZ: standard DSMZ52 medium.
