# Supplementary material for: Upgrading syngas fermentation effluent using Clostridium kluyveri in a continuous fermentation
Source: Biotechnol Biofuels. 2017 Mar 29;10:83. doi: 10.1186/s13068-017-0764-6 (PMC5372331; doi:10.1186/s13068-017-0764-6)
Supplement: Supplementary file 3 — Additional file 3. n-Caprylic acid concentrations during batch experiments; Table S1 with heading and explanation. [file 13068_2017_764_MOESM3_ESM.docx]

## *n*-Caprylic acid concentrations during batch experiments

| **Condition** | **Final concentration (mM)** |
| --- | --- |
| DSMZ | 1.04 ± 0.11 |
| SGP- | 0.16 ± 0.01 |
| SGPT- | 0.83 ± 0.34 |
| SGPT+ | 0.53 ± 0.09 |
| SGM- | 2.19 ± 0.34 |
| SGMT- | 2.07 ± 0.11 |
| P- | 0 |
| M- | 1.52 ± 0.23 |

Table S1 – Concentration of *n*-caprylic acid for each medium type at the end of the 14-day batch experiment. The mean of 3 samples and the standard deviation on the measurement are shown.
