# Supplementary material for: Upgrading syngas fermentation effluent using Clostridium kluyveri in a continuous fermentation
Source: Biotechnol Biofuels. 2017 Mar 29;10:83. doi: 10.1186/s13068-017-0764-6 (PMC5372331; doi:10.1186/s13068-017-0764-6)
Supplement: Supplementary file 4 — Additional file 4. Visual observation of the sporulation behavior of C. kluyveri; Figure S2 with heading and explanation. [file 13068_2017_764_MOESM4_ESM.docx]

## Visual observation of the sporulation behavior of *C. kluyveri*

**
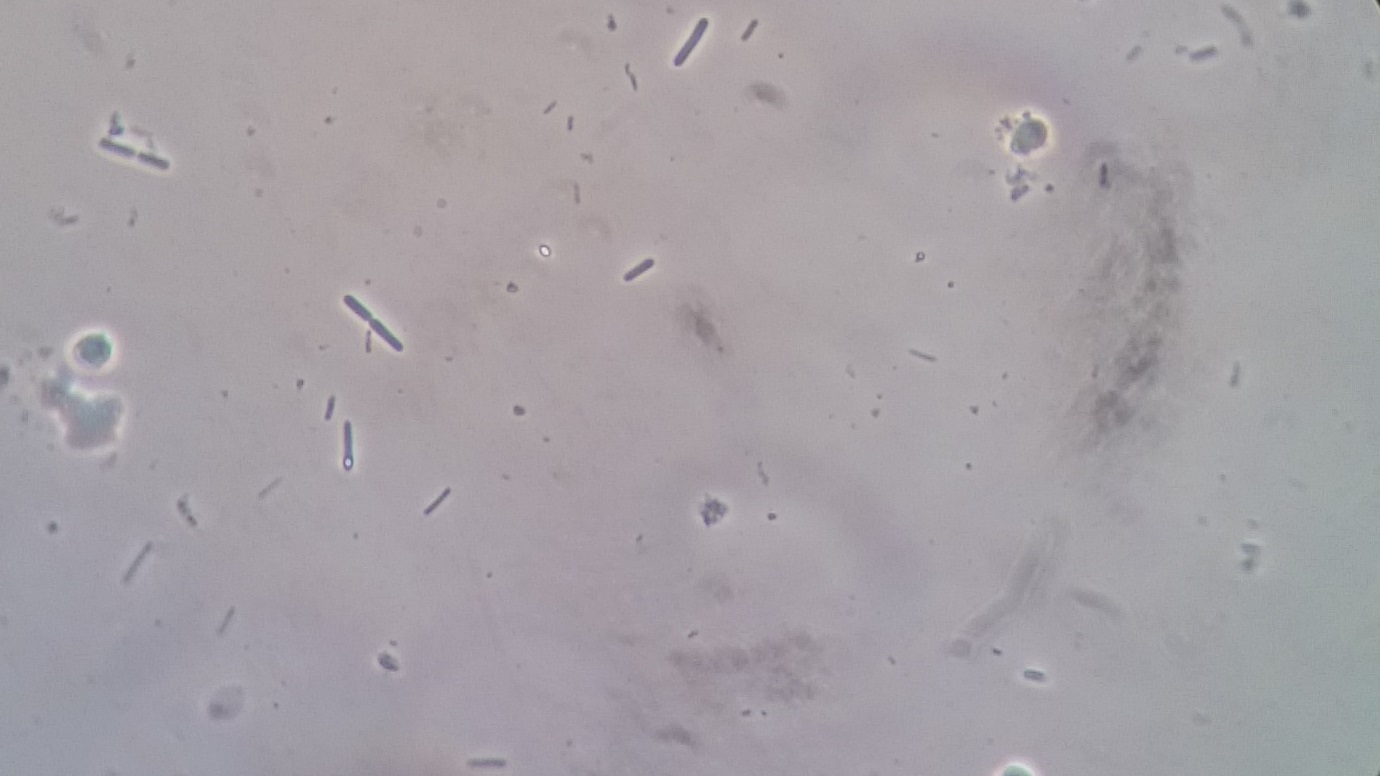
**
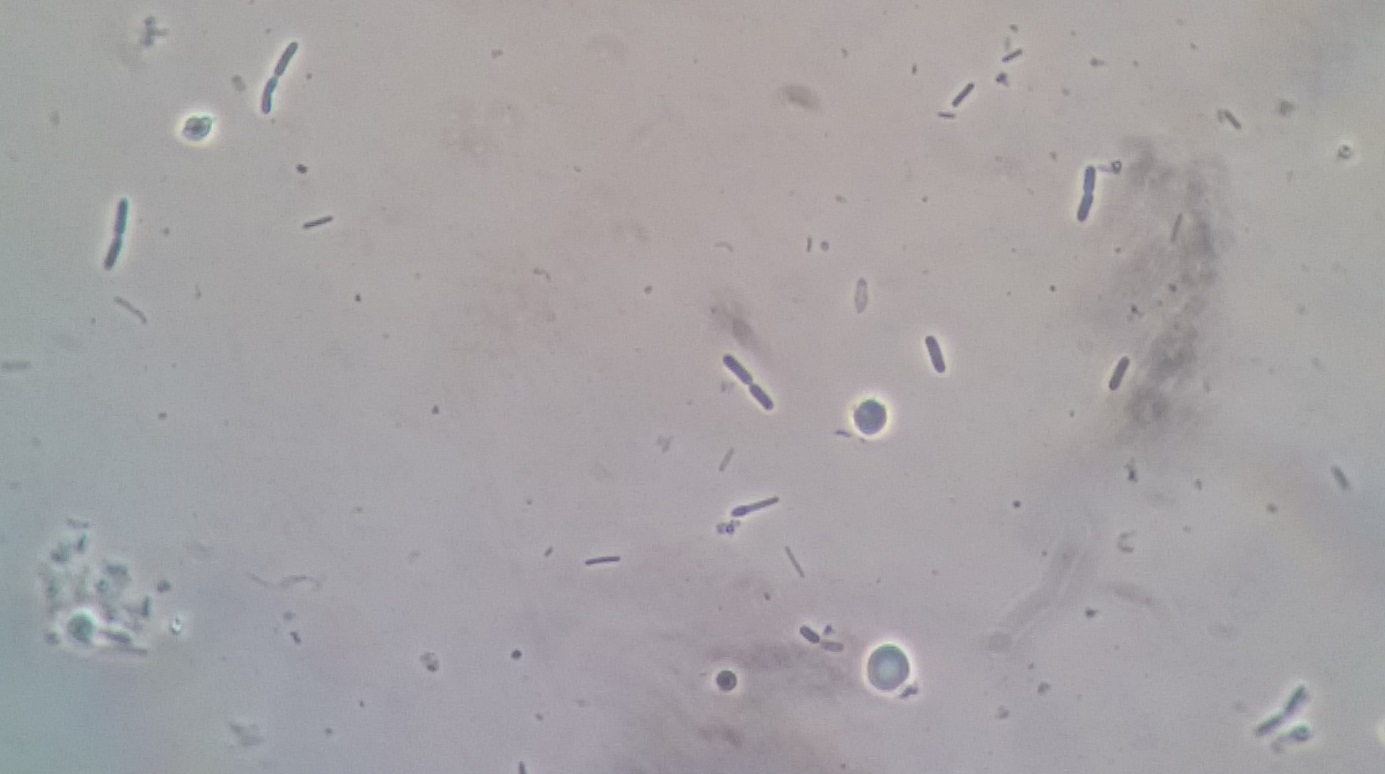


*C. kluyveri*, dividing cells

*C. kluyveri*, outgrowing spore

Outgrown spore, new cell

**B**

**A**

*C. kluyveri*, dividing cells

Spore formed inside cell

spore

**Figure S2 - Light microscopy pictures of bioreactor BP (with pertraction) on day 64 to illustrate the different stages of sporulation of *C. kluyveri*. Photo A: Actively dividing cells; spore being formed inside a cell; free spore. Photo B: Actively dividing cells; outgrowing spore; new cell (from outgrown spore).**
