# Supplementary material for: Upgrading syngas fermentation effluent using Clostridium kluyveri in a continuous fermentation
Source: Biotechnol Biofuels. 2017 Mar 29;10:83. doi: 10.1186/s13068-017-0764-6 (PMC5372331; doi:10.1186/s13068-017-0764-6)
Supplement: Supplementary file 5 — Additional file 5. Optical density; Figure S3 with heading and explanation. [file 13068_2017_764_MOESM5_ESM.docx]

## Optical density

The bioreactor with pertraction (BP) consistently had a lower OD than the bioreactor without pertraction (BNP) (**Figure S3**). In the bioreactor without pertraction, the OD was influenced by the formation of a black precipitate. This precipitate dissolved at lower pH; as such, no precipitate was formed in the bioreactor with pertraction, operating at pH 5.50 or 6.00. In batch experiments precipitates were not formed. From day 80 of the operating period, the validity of OD measurements was checked by dissolving the precipitate in acid. Viability of the cells was checked with microscopy. From then on, OD was always measured after dissolving the precipitate. A short-circuit was furthermore detected in the bioreactor with pertraction (BP), which caused washout of biomass.


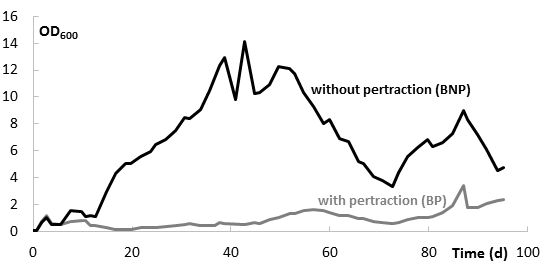


Figure S3 - Optical density (measured at 600 nm) for both bioreactors during the operating period.
