# Supplementary material for: Upgrading syngas fermentation effluent using Clostridium kluyveri in a continuous fermentation
Source: Biotechnol Biofuels. 2017 Mar 29;10:83. doi: 10.1186/s13068-017-0764-6 (PMC5372331; doi:10.1186/s13068-017-0764-6)
Supplement: Supplementary file 6 — Additional file 6. Word document (.docx); Concentrations of carboxylic acids in the bioreactor broth; Figure S4 and S5 with headings and explanations. [file 13068_2017_764_MOESM6_ESM.docx]

## 6. Concentrations of carboxylic acids in the bioreactor broth

Carboxylic acid concentrations for the bioreactor with (BP, **Figure S4**) and without (BNP, **Figure S5**) pertraction. In the bioreactor with pertraction (BP), carboxylic acids were extracted from day 11 of the operating period when the extraction modules were switched on. Part of the produced acids were extracted (mainly the MCCAs). Due to the higher pH in the bioreactor (6 instead of 5.5), and problems with the pertraction unit, the extraction efficiency remained below the anticipated levels (**main text** and **Table 3**).


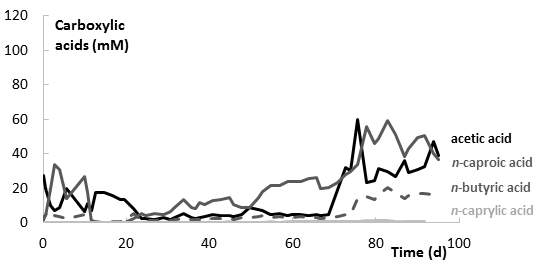


Figure S4 - Concentrations of the carboxylic acids in the bioreactor broth for the bioreactor with pertraction (BP, operating pH 6 from day 20). The low broth concentrations are due to extraction of the products in the pertraction system.


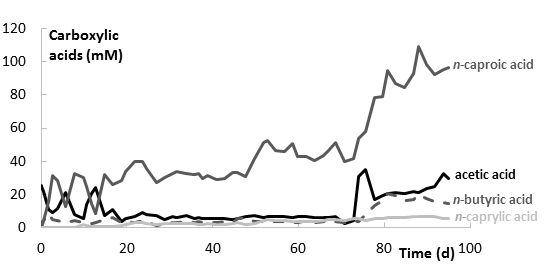


Figure S5 - Concentrations of the carboxylic acids in the bioreactor broth for the bioreactor without pertraction (BNP, operating at pH 7).
