# Supplementary material for: Upgrading syngas fermentation effluent using Clostridium kluyveri in a continuous fermentation
Source: Biotechnol Biofuels. 2017 Mar 29;10:83. doi: 10.1186/s13068-017-0764-6 (PMC5372331; doi:10.1186/s13068-017-0764-6)
Supplement: Supplementary file 7 — Additional file 7. Ethanol concentrations in the bioreactors; Figure S6 with heading and explanation. [file 13068_2017_764_MOESM7_ESM.docx]

## Ethanol concentrations in the bioreactors


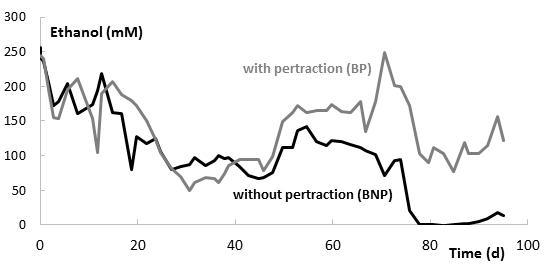


Figure S6 - Ethanol concentration in the bioreactor broth during the operating period for BP and BNP.
