# Supplementary material for: Upgrading syngas fermentation effluent using Clostridium kluyveri in a continuous fermentation
Source: Biotechnol Biofuels. 2017 Mar 29;10:83. doi: 10.1186/s13068-017-0764-6 (PMC5372331; doi:10.1186/s13068-017-0764-6)
Supplement: Supplementary file 8 — Additional file 8. Conversion efficiency; Figure S7 with heading and explanation. [file 13068_2017_764_MOESM8_ESM.docx]

## Conversion efficiency

Conversion efficiencies were calculated on a carbon basis. The ethanol and acetic acid were considered as input carbon, while the produced *n*-butyric acid, *n*-caproic acid, and *n*-caprylic acid as output carbon. Biomass production and CO_2_ were not taken into account for the mass balance.


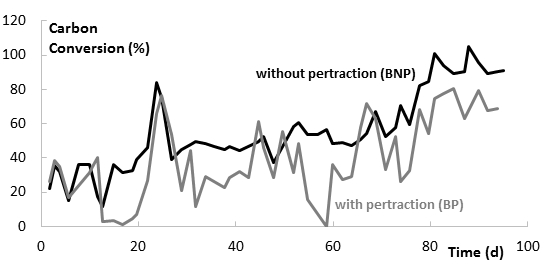


Figure S7 - Conversion of carbon in the substrate (ethanol and acetic acid) to carbon in products (*n*-butyric acid, *n*-caproic acid, and *n*-caprylic acid), which was expressed as carbon conversion efficiency (%).
