# Supplementary material for: Upgrading syngas fermentation effluent using Clostridium kluyveri in a continuous fermentation
Source: Biotechnol Biofuels. 2017 Mar 29;10:83. doi: 10.1186/s13068-017-0764-6 (PMC5372331; doi:10.1186/s13068-017-0764-6)
Supplement: Supplementary file 9 — Additional file 9. Sequencing data; Figure S8 with heading and explanation. [file 13068_2017_764_MOESM9_ESM.docx]

## Sequencing data


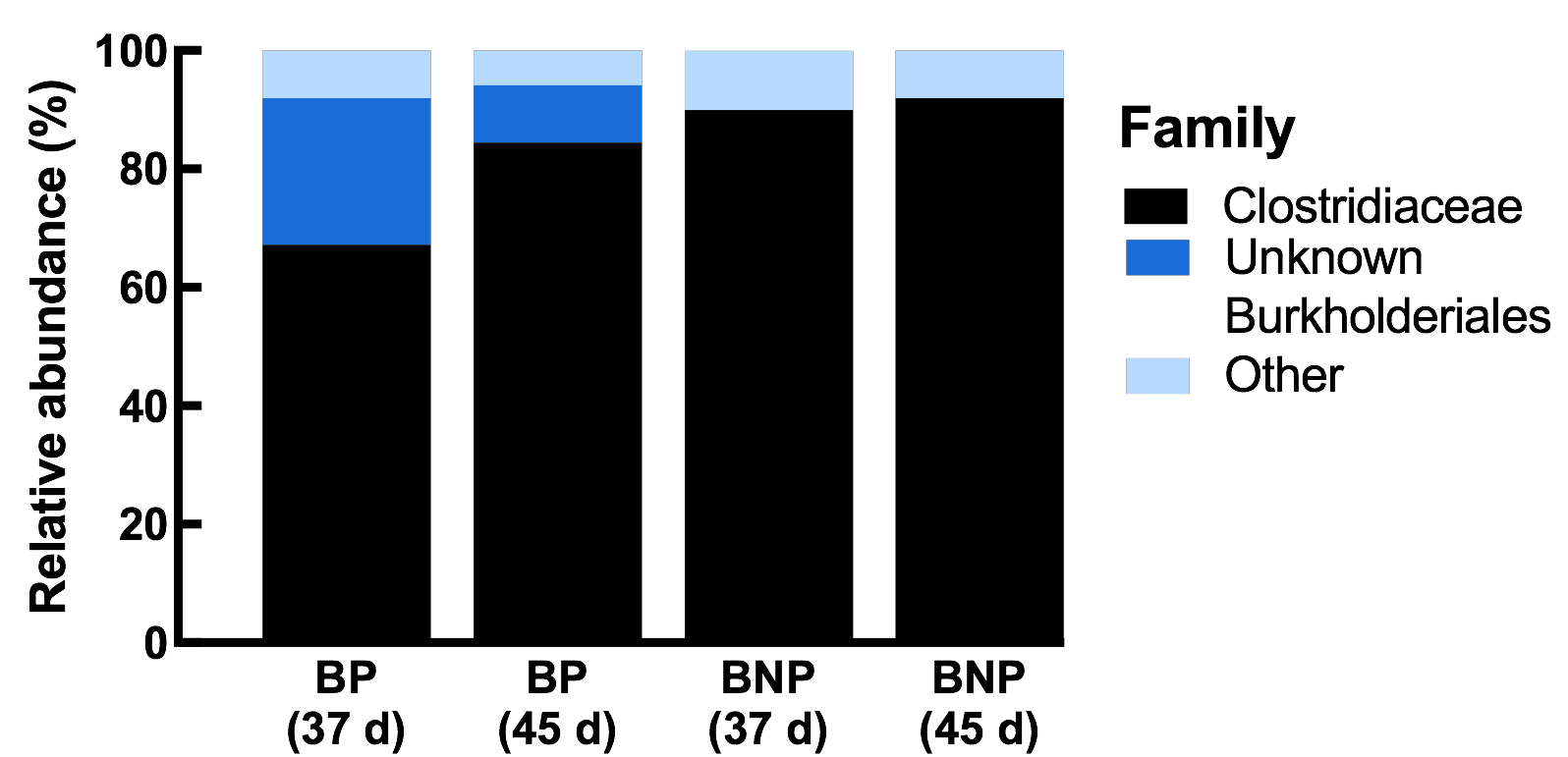


Figure S8 - Bacterial relative abundance distribution within each bioreactor community. Each vertical bar represents one sample taken at the time point indicated. Taxonomic abundance is displayed at the Family level. Only two major taxa are dominant: Clostridiaceae and an unknown Burkholderiales.
